# Lipidomics Reveals Multiple Pathway Effects of a Multi-Components Preparation on Lipid Biochemistry in ApoE*3Leiden.CETP Mice

**DOI:** 10.1371/journal.pone.0030332

**Published:** 2012-01-23

**Authors:** Heng Wei, Chunxiu Hu, Mei Wang, Anita M. van den Hoek, Theo H. Reijmers, Suzan Wopereis, Jildau Bouwman, Raymond Ramaker, Henrie A. A. J. Korthout, Marco Vennik, Thomas Hankemeier, Louis M. Havekes, Renger F. Witkamp, Elwin R. Verheij, Guowang Xu, Jan van der Greef

**Affiliations:** 1 Department of Earth, Environmental and Life Science, TNO, Zeist, The Netherlands; 2 Sino-Dutch Centre for Preventive and Personalized Medicine, Zeist, The Netherlands; 3 Division of Analytical Bioscience, Leiden/Amsterdam Center for Drug Research, Leiden University, Leiden, The Netherlands; 4 CAS Key Laboratory of Separation Science for Analytical Chemistry, Dalian Institute of Chemical Physics, Chinese Academy of Sciences, Dalian, China; 5 SU BioMedicine, Zeist, The Netherlands; 6 Metabolic Health Research, TNO, Leiden, The Netherlands; 7 Fytagoras B.V, Leiden, The Netherlands; 8 Netherlands Metabolomics Centre, Leiden University, Leiden, The Netherlands; 9 Department of Endocrinology and Cardiology, Leiden University Medical Centre, Leiden, The Netherlands; 10 Division of Human Nutrition, Wageningen University, Wageningen, The Netherlands; University of Tor Vergata, Italy

## Abstract

**Background:**

Causes and consequences of the complex changes in lipids occurring in the metabolic syndrome are only partly understood. Several interconnected processes are deteriorating, which implies that multi-target approaches might be more successful than strategies based on a limited number of surrogate markers. Preparations from Chinese Medicine (CM) systems have been handed down with documented clinical features similar as metabolic syndrome, which might help developing new intervention for metabolic syndrome. The progress in systems biology and specific animal models created possibilities to assess the effects of such preparations. Here we report the plasma and liver lipidomics results of the intervention effects of a preparation SUB885C in apolipoprotein E3 Leiden cholesteryl ester transfer protein (ApoE*3Leiden.CETP) mice. SUB885C was developed according to the principles of CM for treatment of metabolic syndrome. The cannabinoid receptor type 1 blocker rimonabant was included as a general control for the evaluation of weight and metabolic responses.

**Methodology/Principal Findings:**

ApoE*3Leiden.CETP mice with mild hypercholesterolemia were divided into SUB885C-, rimonabant- and non-treated control groups. SUB885C caused no weight loss, but significantly reduced plasma cholesterol (−49%, *p*<0.001), CETP levels (−31%, *p*<0.001), CETP activity (−74%, *p*<0.001) and increased HDL-C (39%, *p*<0.05). It influenced lipidomics classes of cholesterol esters and triglycerides the most. Rimonabant induced a weight loss (−9%, *p*<0.05), but only a moderate improvement of lipid profiles. *In vitro*, SUB885C extract caused adipolysis stimulation and adipogenesis inhibition in 3T3-L1 cells.

**Conclusions:**

SUB885C, a multi-components preparation, is able to produce anti-atherogenic changes in lipids of the ApoE*3Leiden.CETP mice, which are comparable to those obtained with compounds belonging to known drugs (e.g. rimonabant, atorvastatin, niacin). This study successfully illustrated the power of lipidomics in unraveling intervention effects and to help finding new targets or ingredients for lifestyle-related metabolic abnormality.

## Introduction

The incidence of lifestyle-related cardiovascular and metabolic health complications, often collectively named metabolic syndrome, continues to increase world-wide [1], [2]. Although the major risk factors, including a sedentary lifestyle, overweight, unfavorable dietary habits and smoking are essentially modifiable, lifestyle measures often prove difficult and disappointing on the longer term. Because of the complex and multi-factorial manifestations of the metabolic syndrome, pharmacological strategies for primary prevention are increasingly focusing on the use of low-dose drug combinations. An example is the development of “polypill” concepts with a statin, one or more anti-hypertensive compounds and acetylsalicylic acid to reduce risks for cardiovascular disease in middle-aged individuals [3], [4]. At the same time it has been shown that dietary measures may be of comparable efficacy. Such a “polymeal” could provide a “more natural, safer and probably tastier alternative” than a polypill [5]. New insights and leads for dietary prevention or intervention can also be acquired from other healthcare systems like Chinese Medicine (CM). In CM [6], the gap between food and drugs has always been small, and nutrition is seen as a normal part of prevention and healthcare. A remarkable high number of preparations have been handed down over the centuries with documented activity related to clinical features of what is now described as metabolic syndrome. The possibilities to analyze the subtle and multiple-pathway effects of such preparations have increased by the developments in systems biology-based metabolomics and specific animal models [7]. Here we report the plasma and liver lipidomic analysis of the effects of a CM preparation, SUB885C, in apolipoprotein E3 Leiden cholesteryl ester transfer protein (ApoE*3Leiden.CETP) transgenic mice. SUB885C is a multi-components preparation developed according to the principles of CM containing eight natural ingredients. The core formula is used in China for treatment of metabolic syndrome and early stage type 2 diabetes with obesity. A SUB885C intervention study [7] in prediabetic ApoE*3 Leiden mice has shown that SUB885C significantly improved insulin sensitivity as compared with non-treated controls. Meanwhile, several other anti-inflammatory and metabolic effects of the active ingredients in SUB885C have been reported [8]–[12]. Therefore, we hypothesized that SUB885C exerts a multi-target activity on lipid metabolism and insulin sensitivity. To investigate this, a parallel controlled intervention study was designed with female ApoE*3Leiden.CETP transgenic mice [13]–[16] showing mild hypercholesterolemia and overweight. The ApoE*3Leiden.CETP mouse model is obtained by cross-breeding ApoE*3Leiden mice with mice expressing human CETP. It has been shown to respond in a human-like manner to both lipid-lowering and high density lipoprotein cholesterol (HDL-C) raising interventions [13]–[19]. Outcome parameters included body weight, food intake, plasma lipids and lipoproteins, and lipidomics of plasma and liver. Lipidomics measures all or subsets of lipids and provides a thorough perspective to study intervention induced lipid changes and metabolism in the complex biological system [7], [20].The cannabinoid receptor type 1 (CB1) blocker rimonabant was included as a general control for the evaluation of weight and metabolic responses in the study. To further explore our findings, cell-based assays in 3T3-L1 adipocytes focusing on adipolytic and adipogenic activities of SUB885C were performed.

## Materials and Methods

### Ethics statement

The experiments were performed according to the rules set by the Netherlands Law on Animal Experiments and approved by the Institutional Ethical Committee on Animal Care and Experimentation (Dierexperimenten Commissie DEC of Netherlands Organization for Applied Scientific Research, Zeist, the Netherlands) with a permit number of DEC2489.

### Materials and chemicals

#### Materials used for intervention studies

SUB885C was provided by SU Biomedicine, The Netherlands. SUB885C consists of eight natural ingredients: *Fructus Crataegi* (Shan Zha), *Folium Nelumbinis* (He Ye), *Folium Apocyni* (Luo Bu Ma Ye), *Flos Rosae rugosae* (Mei Gui Hua), *Radix et Rhizoma Rhei* (Da Huang), *Depuratum mirabilitum* (Mang Xiao, also known as mirabilite or Glauber's salt), *Thallus Sargassi* (Hai Zao*)*, and honey fried *Radix Glycyrrhizae* (Gan Cao). Above dry and sliced compounds were used for a water based decoction. After decoction and water solvent was dried into solid phase, it was used as the preparation for the intervention study. For the quality control of the preparation, the quantities of defined chemical markers were used for assessment according to pharmacopeia guideline.

#### Chemicals and lipid internal standards

Synthetic lipid standards including 1-heptadecanoyl-2-hydroxy-*sn*-glycero-3-phosphocholine (LPC-17∶0), 1-nonadecanoyl-2-hydroxy-*sn*-glycero-3-phosphocholine (LPC-19∶0), 1,2-dipentadecanoyl-*sn*-glycero-3-phosphatidylethanolamine (PE-30∶0), 1,2-diheptadecanoyl-*sn*-glycero-3-phosphoethanolamine (PE-34∶0), 1,2-diheptadecanoyl-*sn*-glycero-3-phospholcholine (PC-34∶0) and 1,2-dinonadecanoyl-*sn*-glycero-3-phospholcholine (PC-38∶0) were purchased from Avanti Polar Lipids, Inc. (Alabaster, AL, USA). 1,2,3-tripentadecanoylglycerol (TG-45∶0) and 1,2,3-triheptadecanoylglycerol (TG-51∶0) were obtained from Sigma-Aldrich Chemie B.V. (Zwijndrecht, The Netherlands). Ultra liquid chromatography-mass spectrometry (Ultra LC-MS) grade of acetonitrile (ACN), methanol (MeOH), isopropanol (IPA) and water as well as LC–MS grade of dichloromethane (CH_2_Cl_2_) were purchased from Biosolve (Valkenswaard, The Netherlands). Phosphate Buffered Saline (PBS), and Hanks Balanced Salt Solution (HBBS) were supplied by Gibco. Isoproterenol, 10% sterile bovine serum albumin (BSA) solution, methanol and dimethyl sulfoxide (DMSO) were supplied by Sigma Aldrich. Plastic ware for tissue culture was supplied by Greiner Bio-One. The adipolysis assay kit was purchased fom Chemicon Int. (Temecula, CA).

### Intervention studies on mice

Twenty-four female ApoE*3Leiden.CETP transgenic mice (age 6–10 weeks) were obtained from specific pathogen free (SPF) breading stock (TNO, Leiden, The Netherlands). The animals were fed a semi-synthetic modified Western-type diet (Hope Farms, The Netherlands), containing 0.2% cholesterol (Cho), 15% saturated fat and 40% sucrose as described by Nishina et al. [21], for a run-in period of 4 weeks to get a mild hypercholesterolemia (plasma Cho levels of 14–18 mmol/L) and body weight increase. Following this run-in period, mice were matched on body weight, plasma Cho and triglycerides (TG) levels (after 4 hours fasting) and divided in three groups of eight animals each (non-treated control, rimonabant- and SUB885C- treated). Preparations for intervention were given orally as admix to a Western-type diet for 4 weeks. Briefly, mice received the Western-type diet, without or with either SUB885C (SU Biomedicine, The Netherlands) at a concentration of 2% or rimonabant (Sanofi-Aventis, The Netherlands) at 10 mg/kg body weight/day. Flavoured sugar lumps were added to the diet of all groups to mask possible tastes of intervention preparations. Body weight per mouse and food intake per cage was measured at intervention day 0, 2, 3, 4, 9, 11, 14, 21 and 28. Blood was collected at the start of the intervention (week 0) and just before sacrifice of the mice (week 4) via tail vein bleeding using CB 300 LH microvettes (Sarstedt, Nümbrecht, Germany). Plasma samples were collected by centrifugation of the blood samples for 10 min at 6000 rpm at 4°C. At the end of the intervention (week 4), animals were sacrificed with rapid asphyxiation using CO_2_. Livers were dissected on ice and samples were weighted and snap-frozen in liquid nitrogen. Both plasma and liver samples were stored at −80°C until use.

### Adipocyte studies

The 3T3-L1 preadipocyte cell line (ATCC) was used to study possible direct lipolysis or adipogenetic activity by SUB885C. The 3T3-L1 cells were cultured and differentiated from pre-adipocytes into full grown adipocytes as described by Niwano et al. [22]. Three hundred micrograms of SUB885C was extracted with 4 ml methanol. After evaporation of the methanol, the extract was dissolved in 100 µl DMSO. To study adipolysis the growth medium was removed from the cells and cells were washed twice with 1 ml HBSS per well. Then, to each well 250 µl HBSS was added containing 2% BSA and 2.5 µl of the diluted SUB885C extracts in DMSO. As a positive control, 2.5 µl of 1 mM isoproterenol in DMSO was added (final concentration 10 µM isoproterenol). For the negative controls, either 2.5 µl DMSO (DMSO control) or nothing (blank) was added. After 3 h incubation glycerol release was measured with a commercial adipolysis assay kit (Chemicon Int., Temecula, CA). For the adipose conversion assay the addition of SUB885C started at the initiation of the cell differentiation. Together with the differentiation medium, SUB885C extract was added at different dilutions. At every medium replacement, fresh extract was added as well. After nine days of differentiation, the adipose conversion of the cells was analyzed by measuring the amount of fat produced. This was done by staining the fat with Oil Red O as described by Ramírez-Zacarías et al. [23].

### Plasma biochemical analyses and lipoprotein profile analysis

Plasma Cho, TG, HDL-C, lipoprotein profiles, CETP levels and activities and alanine aminotransferase (ALT) were measured at week 0 and week 4. Plasma total cholesterol (TC) and TG concentrations were determined in each animal using enzymatic kits (Roche Molecular Biochemicals, Indianapolis, Ind, USA). Pooled lipoprotein profiles were measured by fast performance liquid chromatography (FPLC) using an AKTA apparatus (Amersham Biosciences). TC, TG and phospholipid levels were measured in the fractions of freshly obtained samples. Phospholipids were determined in the FPLC fractions using a phospholipids B kit (Instruchemie Co., The Netherlands). Plasma HDL was determined by quantification of HDL-C in plasma after precipitation of apoB-containing lipoproteins. Thus, 10 µl of heparin (LEO Pharma, The Netherlands) and 10 µl of 0.2 mol/L MnCl_2_ were added to 20 µl plasma, and mixtures were incubated for 20 min at room temperature and centrifuged for 15 minutes at 13000 *g* at 4°C. Plasma ALT was measured in pooled samples using a Boehringer Reflotron system. CETP levels were measured per mouse using RB-CETP kits from Roar Biomedical, Inc. Endogenous CETP activity was measured as described before [24]. Briefly, ^3^H cholesterol was equilibrated for 24 h with plasma cholesterol at 4°C followed by incubation at 37°C for 3 h. Subsequently, apoB-containing lipoproteins were precipitated by addition of heparin/MnCl_2_. Lipids were extracted from the precipitate and labeled cholesteryl esters were separated from labeled unesterified cholesterol on silica columns and assayed by liquid scintillation counting.

### Lipidomics analyses

#### Lipid extraction for plasma samples

Plasma samples were thawed to room temperature and extracted with 2∶1 of CH_2_Cl_2_/MeOH as described previously [25]. Briefly, 30 µl of heparin plasma was placed in a 2 ml vial (Eppendorf, Hamburg, Germany). Thirty microliters of the internal standard (IS) mixture consisting of LPC-19∶0, PE-30∶0, PC-38∶0 and TG-45∶0 with corresponding concentrations of 30, 30, 150 and 60 µg/ml was first added, followed by 190 µl of MeOH and then 380 µl of CH_2_Cl_2_. The mixture was thoroughly vortexed both before and after CH_2_Cl_2_ addition. Afterwards, 120 µl of water was added and thoroughly vortexed. After centrifuging for 10 min at 6000 g at 10°C, 300 µl of the lower organic phase was transferred into a new autosampler vial and stored at −20°C until analysis. For LC-MS analysis, 25 µl of the lipid extract was diluted with 475 µl ACN/IPA/water 65/30/5 (v/v/v) and 10 µl was injected.

#### Lipid extraction for liver samples

The frozen liver samples stored at −80°C were lyophilized and ground into powder. Ten milligrams of liver powder was weighed in a clean 1.5 ml eppendorf vial for the subsequent lipid extraction. Liver lipid extraction was achieved by liquid-liquid extraction (LLE) with a CH_2_Cl_2_/MeOH mixture (2∶1, v/v) based on the method of Bligh and Dyer [26] with some modifications. Briefly, 60 µl of IS were added to 10 mg of dry liver powder followed by addition of 180 µl of MeOH containing 0.02% antioxidant butylated hydroxytoluene (BHT), and then 360 µl of CH_2_Cl_2_ was added. The mixture was vortexed for 1 min both before and after CH_2_Cl_2_ addition. Afterwards, the resulting suspension was placed for 5 min in an ultrasonic bath at 4°C and then put in a shaker followed by 45 min incessantly shaking at 4°C. Thereafter 10 min centrifugation at a rotation speed of 6000 *g* at 10°C was applied before 500 µl of the supernatant was transferred into a new 1.5 ml eppendorf vial. One hundred microliters of 0.9% NaCl was subsequently added to the supernatant to get a two-phase system where most of the lipids were in the lower organic phase. After being centrifuged at 2000 *g* at 10°C for 10 min, a total of 300 µl of lipid extract was collected from the bottom organic phase. The final extract was diluted 40 times by injection solvent as described previously [25] and then 10 µl was injected for LC-MS analysis.

### LC-MS lipid profiling

LC-MS analysis was performed on a hybrid liquid chromatography-linear ion trap-Fourier transform ion cyclotron resonance-mass spectrometric system (LC-FTMS) consisting of a Surveyor HPLC MS pump and an autosampler (Thermo Fisher) equipped with an Ascentis Express C8 2.1×150 mm (2.7 µm particle size) column (Sigma-Aldrich Chemie B.V., Zwijndrecht, The Netherlands). The LC-MS method used has been described previously by Hu et al. [25]. With this method, seven different lipid classes including both polar lipids such as lyso-phosphatidylcholine (LPC), lyso-phosphoethanolamine (LPE), phosphatidylcholine (PC), phosphoethanolamine (PE), sphingomyelin (SPM) and non-polar lipids such as cholesterol esters (ChEs) and TG were eluted from the column ionized with electrospray ionization in the positive ion mode. The MS detection was performed in the full scan mode with a range of mass to charge ratio (m/z) 400–1500. The identification of the detected lipid peaks was performed as described previously [25] and the current accurate mass data acquired by FT and linear-ion-trap MS/MS fragmentation. For those peaks without MS/MS fragmentations, identification was based on the observed accurate m/z and the relative retention times of specific m/z peaks. Forty-eight plasma samples (week 0 and week 4) and 24 liver samples (week 4) from ApoE*3.CETP transgenic mice of three groups were prepared in duplicate and injected once according to the procedures described above. The performance of the applied lipid profiling platform was assessed through the repeated analysis of the quality control (QC) samples [27]. The QC samples, used to monitor the LC-MS response in time, were prepared by pooling aliquots of 48 plasma samples for plasma lipidomics and 24 liver samples for liver lipidomics respectively to represent the full biochemical diversity of the study samples and allow the calculation of the analytical precision for all lipids measured. The QC sample data is also used to correct systematic errors such as batch to batch response differences by a single point calibration model [28], [29]. Ten QC plasma samples and 5 QC liver samples were processed exactly in the same way as the study samples. In total 106 plasma samples including 96 study samples and 10 QC samples and 53 liver samples including 48 study samples and 5 QC samples were injected into the LC-MS system. The study samples were randomly analyzed and the QC samples were placed at regular intervals in the analysis sequence (one QC after every 10 samples). Furthermore, method performance was carefully monitored using multiple IS and duplicate analysis of QC samples. Of note, plasma and liver samples were analyzed separately.

### Lipidomics data processing

In total, 140 plasma lipid peaks and 137 hepatic lipid peaks were identified and selected as target lipids based on retention time and m/z of peaks processed by LC-Quan 2.5 (Thermo Fisher). The peak detection algorithm was used for peak integration using the following parameters: ICIS; smoothing points, 7; window, 30 s; view width, 3 min; baseline, 40; area noise factor, 5; peak noise factor, 1045. Data for each mouse was normalized for the recovery of the IS for injection. Batch to batch differences in the data were removed by synchronizing the medians values of the QC samples per batch. Data was used only if the duplicates corresponded after visual inspection and the duplicates were averaged.

### Statistical data analysis

One mouse (number 3733) in the control group was excluded from data analyses, because it did not respond to the diet during the run-in period and failed the inclusion criteria for hypercholesterolemia. Univariate data analyses were done by SPSS 17.0 and the results are presented as means ± standard deviation (SD) unless indicated otherwise. Statistical differences of biochemical parameters and lipidomics regulation at the end of the intervention of the three groups were analyzed parametrically by one-way analysis of variance (ANOVA). Data were log transformed if homogeneity of variance assumption was rejected. The Dunnett post hoc method was used to identify which treatment was significantly different from the control. One-tailed and two-tailed tests were used for biochemistry parameters and the lipidomics data respectively, with *p* values <0.05 as statistical significance. To correct for false positives, the multiple test correction (MTC) of Benjamini and Hochberg false discovery rate (FDR) analysis was applied to adjust *p* values derived from the univariate results of the lipidomics data. To avoid the possibility that a few high-intensity variables dominate the final results [30], plasma and liver lipidomics datasets were autoscaled per variable, meaning that first the means of each variable were subtracted, and then all variables were divided by their SD. To find treatment dependent lipidomics grouping effects, both lipidomics datasets were analyzed by Principal Component Analysis (PCA) [31] in MATLAB (version 7.7.0471, the Mathworks) with the PLS toolbox (version 5.0.3, Eigenvector Research, InC.). PCA was used to find patterns in the data such as clusters of mice (scores) of non-treated controls and undergoing the treatments; and to identify which lipids contributed most to these clusters (loadings). Partial least squares discriminant analysis (PLS-DA) [32] was further applied to identify the specific metabolites which contribute most to discriminate between the subtypes; and PLS-DA models were validated using double cross validation (DCV) [33]. Week 4 lipidomics were used for data analysis because (1) the study interest is in the effect of SUB885C in the end of the intervention and all baseline parameters are homogeneous among the three groups; (2) liver lipidomics results are only available at week 4. Finally data matrices of 15 mice (7 controls, 8 SUB885C) ×140 lipids for plasma lipidomics and 15 mice ×137 lipids for liver lipidomics were used for multivariate data analysis.

## Results

### SUB885C does not influence food intake or body weight in ApoE*3Leiden.CETP mice

**Figure 1:**
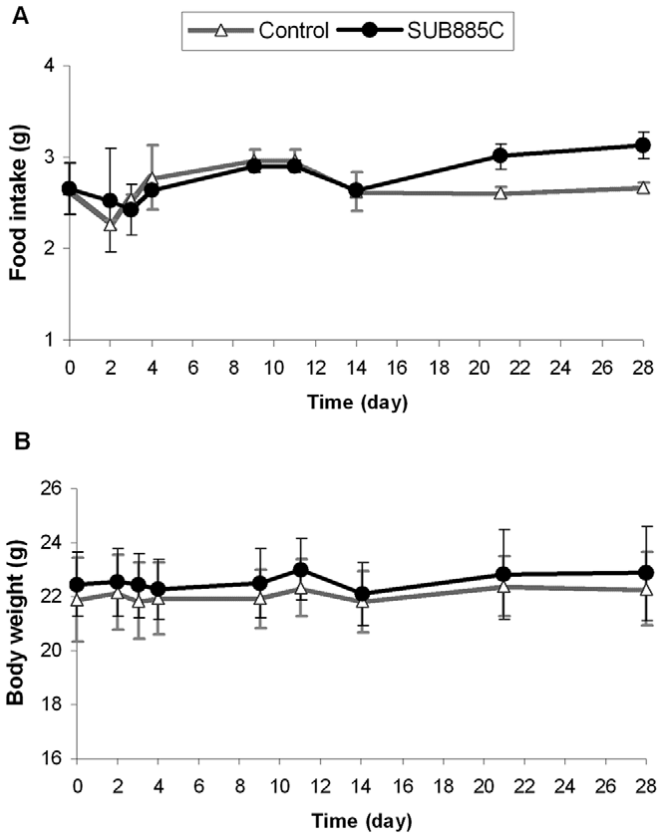
Food intake and body weight. (A) Food intake (per cage) and (B) body weight (per mouse) were recorded and measured at day 0, 2, 3, 4, 9, 11, 14, 21 and 28 for SUB885C-treated mice and control (*p<0.05 vs. control).

Compared to the controls, SUB885C did not have an effect on food intake in ApoE*3Leiden.CETP mice until day 20, and did not affect mean body weight (Figure 1). After day 21, an increase in food intake was observed with SUB885C. According to published results in the same experiment [34], mice treated with rimonabant showed a reduced food intake with a pronounced dip at day 2. A significant reduction of mean body weight of approximately 9% remained visible until the end of the intervention [34].

### SUB885C decreases plasma cholesterol, triglycerides and increases HDL-C

**Figure 2:**
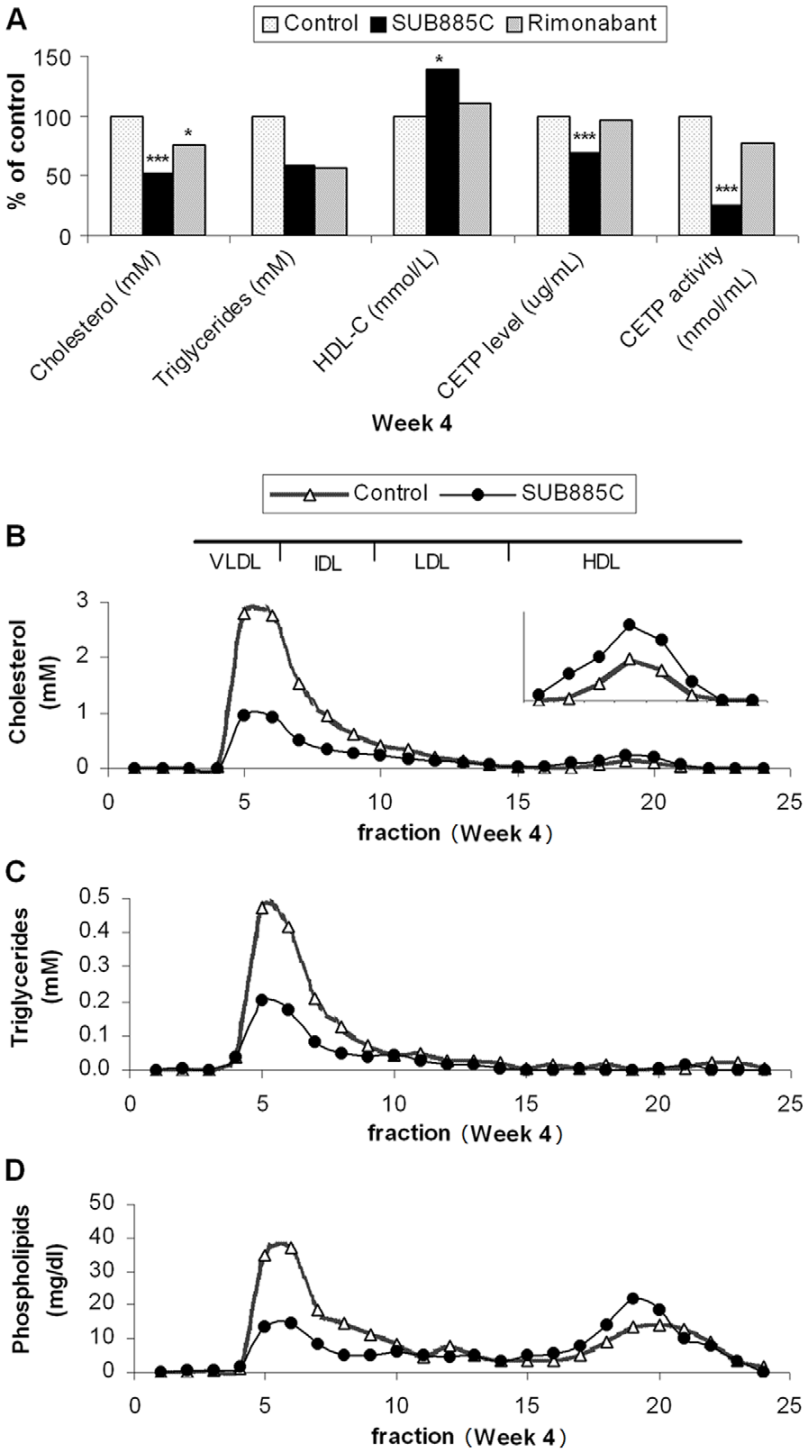
Plasma lipid, CETP and lipoprotein. Plasma concentrations are shown for TC, TG, HDL-C, CETP level and activity (A) of the SUB885C-, rimonabant- and non-treated mice at week 4 (concentrations in the control group are set to be 100% and relative changes of treated groups were illustrated in % compared with the control, *p<0.05, ** p<0.01, *** p<0.001). Alterations of Cho (B), TG (C) and phospholipids (D) in the pooled lipoprotein profiles of the SUB885C- and non-treated mice at week 4. Fractions 4–7 as VLDL; 8–9 as IDL; 10–15 as LDL and 16–23 as HDL.

SUB885C treatment of ApoE*3Leiden.CETP mice caused a significant decrease in plasma Cho by 49% (8±1 mM versus 15±2 mM, *p*<0.001) after 4 weeks as compared to the controls (Figure 2A). During the 4 week intervention period plasma TG levels in both the SUB885C treated group and in control mice were significantly reduced by 67% (1.4±0.5 mM versus 4±2 mM, *p*<0.01) and 46% (2±1 mM versus 4±2 mM, *p*<0.01), respectively (data not shown). At week 4, TG levels in the SUB885C group tended to be lower compared to the control mice (Figure 2A), but this difference did not reach significance (1.4±0.5 mM versus 2±1 mM, *p* = 0.06). Plasma HDL-C in the SUB885C group was significantly increased by 39% (1.1±0.3 mmol/L versus 0.8±0.3 mmol/L, *p*<0.05) as compared to that of the controls in week 4. Lipoprotein fraction analyses showed that SUB885C treatment caused a 3-fold decrease of very low density lipoprotein-cholestol (VLDL-C) and 2.5 fold of VLDL-TGs and VLDL-phospholipids, as compared to those of the control group (Figure 2 B–D). In ApoE*3Leiden.CETP mice, the reduction of plasma TC was caused by the decrease of (V)LDL-C; while the increase of plasma HDL-C was in line with the obvious increase in HDL-C fraction.

Based on previous published results [34], rimonabant treatment of ApoE*3Leiden.CETP mice induced a significant decrease of plasma Cho with 24% (*p*<0.05) when compared with that of the controls. However, there were no significant changes of plasma TG and HDL-C at the end of the intervention. No adverse signs were observed during the study and ALT levels of all three groups were in the reference range.

### SUB885C decreases plasma CETP level and activity

Four weeks of SUB885C intervention (Figure 2A) of ApoE*3Leiden.CETP mice caused a significant decrease in CETP level by 31% (22±2 µg/mL versus 31±4 µg/mL, *p*<0.001) and CETP activity by 74% (24±11 µg/mL versus 91±27 µg/mL, *p*<0.001) as compared to the controls. However, rimonabant did not significantly affect the CETP level or the CETP activity in the same experimental setting [34].

### Lipidomics reveals detailed lipid changes caused by SUB885C

**Figure 3:**
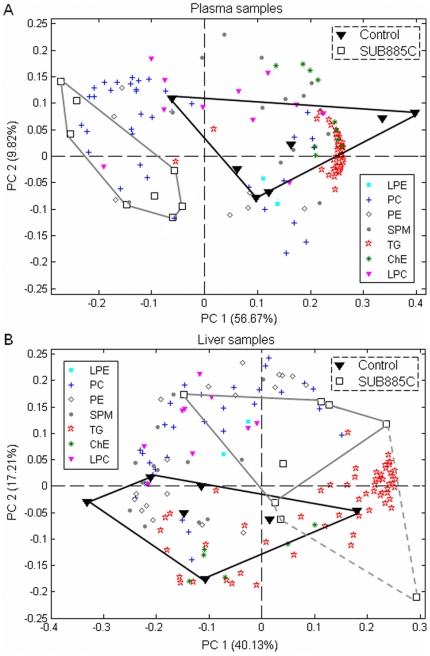
Lipidomics of plasma and liver reveals differences between non-treated and SUB885C-treated mice. Principle component analyses (PCA) of plasma and liver lipidomics datasets were applied to differentiate the non-treated controls (n = 7) and the SUB885C treated mice (n = 8). PCA biplots for (A) plasma samples and (B) liver samples.

Two Principal Components (PCs) were selected for plasma and liver lipidomics. These PCs described 67% and 57% of the total variance of the plasma- and liver-lipidomics, respectively (Figure 3A and B). In both PCA biplots, a separation was observed between control- and SUB885C treated-mice, indicating major changes of measured plasma and liver lipids between two groups. The loadings in the biplots (lipid species represented by colored symbols in Figure 3A and B) indicated that the lipids separating the SUB885C treated-mice from the control mice were TG, ChE and SPM. PLS-DA with DCV was used to further investigate the main discriminating lipids between SUB885C and control groups. The DCV error rates in both plasma and liver were 20%, indicating that 3 out of 15 mice were misclassified. Among 30 main discriminating lipids between the control and SUB885C treated mice, ChEs, SPMs, PCs, TGs contributed most for the separation in plasma while SPMs, PCs, TGs contributed most in liver (Table S1).

Quantitative differences in liver and plasma lipids between the SUB885C treatment group and untreated controls were statistically further analyzed by ANOVA with a two-tailed Dunnett post hoc method. SUB885C caused a significant reduction of plasma lipid classes of SPM, ChE, and TG by −28% (*p*<0.001), −46% (*p*<0.001), and −60% (*p*<0.01) compared to the controls, respectively. It caused a 12% reduction (*p*<0.05) of total liver SPM, without significant influence on total liver TG or ChE lipids.

It was found that 86 (61%) plasma and 22 (16%) liver lipids out of 140 and 137 lipids respectively were significantly changed after the 4-week SUB885C intervention as compared to the controls. Fifteen lipids changed significantly under SUB885C intervention in both plasma and liver, including LPC (18∶2), PC (36∶5), PE (34∶2), SPM (22∶1), SPM (24∶1), ChE (18∶2), ChE (20∶4), TG (50∶1), TG (54∶0), ether TG (TG-O) (50∶0), TG-O (50∶1), TG-O (50∶2), TG-O (52∶1),TG-O (52∶2) and TG-O (58∶2) (Table S2 and S3). The correlation analysis, data with normal distribution by Pearson's and without by Spearman's, was performed to evaluate whether there is a relation between the concentrations of these lipids in plasma and liver. Only TG-O (50∶0) showed a positive correlation between its concentration of plasma and liver; the others showed no correlation (data not shown).

After MTC analysis, 70 out of 86 plasma lipids and 3 out of 22 hepatic lipids remained significant (*p* = 0.00−0.02, *p* values marked in bold) by SUB885C intervention (Table S2 and S3). All three significantly changed hepatic lipids and 68 out of 70 (97%) significantly changed plasma lipids were down regulated by SUB885C treatment. In plasma, SUB885C caused a significant reduction of 80% neutral lipids including ChE and TG, 50% SPM, 17% LPC and 14% PE. Three out of 5 significantly changed plasma PC (14%) by SUB885C was down-regulated (Table 1). In liver, SUB885C caused a significantly reduction of 17% ChE and 3% TG, but no obvious changes in phospholipids. Thus SUB885C induced lipid changes were more prominent in plasma (50%, 70 out of 140, changed lipids) than in liver (2%, 3 out of 137, changed lipids). It affected the lipid classes of TG, ChE and SPM the most, which was in line with PCA results.

**Table 1:** Significantly changed lipids in each lipid class.

Plasma lipidomics			
Lipid class	All measured lipids (n)	Sig. changed lipids	Sig. reduced lipids
		(n, % in each lipid class)	(n, % in each lipid class)
LPC	12	3 (17%)	3 (17%)
LPE	2	0	0
PC	36	5 (14%)	3 (8%)
PE	7	1 (14%)	1 (14%)
SPM	14	7 (50%)	7 (50%)
ChE	10	8 (80%)	8 (80%)
TG	59	47 (80%)	47 (80%)
**Total**	**140**	**70 (50%)**	

Liver lipidomics			
Lipid class	All measured lipids (n)	Sig. changed lipids	Sig. reduced lipids
		(n, % in each lipid class)	(n, % in each lipid class)
LPC	9	0	0
LPE	2	0	0
PC	29	0	0
PE	19	0	0
SPM	13	0	0
ChE	6	1 (17%)	1 (17%)
TG	59	2 (3%)	2 (3%)
**Total**	**137**	**3 (2%)**	

Compared with the results of rimonabant induced lipid changes from the previous publication [34], twenty four plasma lipids including LPC (18∶2), PC (38∶2), SPM (16∶0), ChE (18∶1), TG (46∶0), TG (46∶1), TG (48∶0), TG (48∶1), TG (48∶2), TG (50∶0), TG (50∶1), TG (52∶2), TG (52∶6), TG (54∶2), TG (54∶3), TG (54∶4) TG (56∶3), TG (56∶4), TG (56∶7), TG (58∶3), TG (58∶4), TG (58∶5), TG (58∶6), TG (58∶7) were significantly changed by both SUB885C and rimonabant, all of which were down regulated except for SPM (16∶0) that SUB885C induced a down-regulation while rimonabant a up-regulation. After MTC, only LPC (18∶2) and PC (38∶2) remained significant in both SUB885C and rimonabant groups; while all lipids except for SPM (16∶0), TG (56∶3) and TG (58∶5) still remained significant under SUB885C intervention. Two liver lipids, PE (36∶3) and TG (50∶1), were significantly changed by SUB885C and rimonabant. The former was down-regulated by both treatments while the latter was down-regulated by SUB885C but up-regulated by rimonabant. Both lipids did not hold significance after MTC in SUB885C and rimonabant treatment.

### SUB885C is able to stimulate adipolysis and inhibit adipogenesis *in vitro*

**Figure 4:**
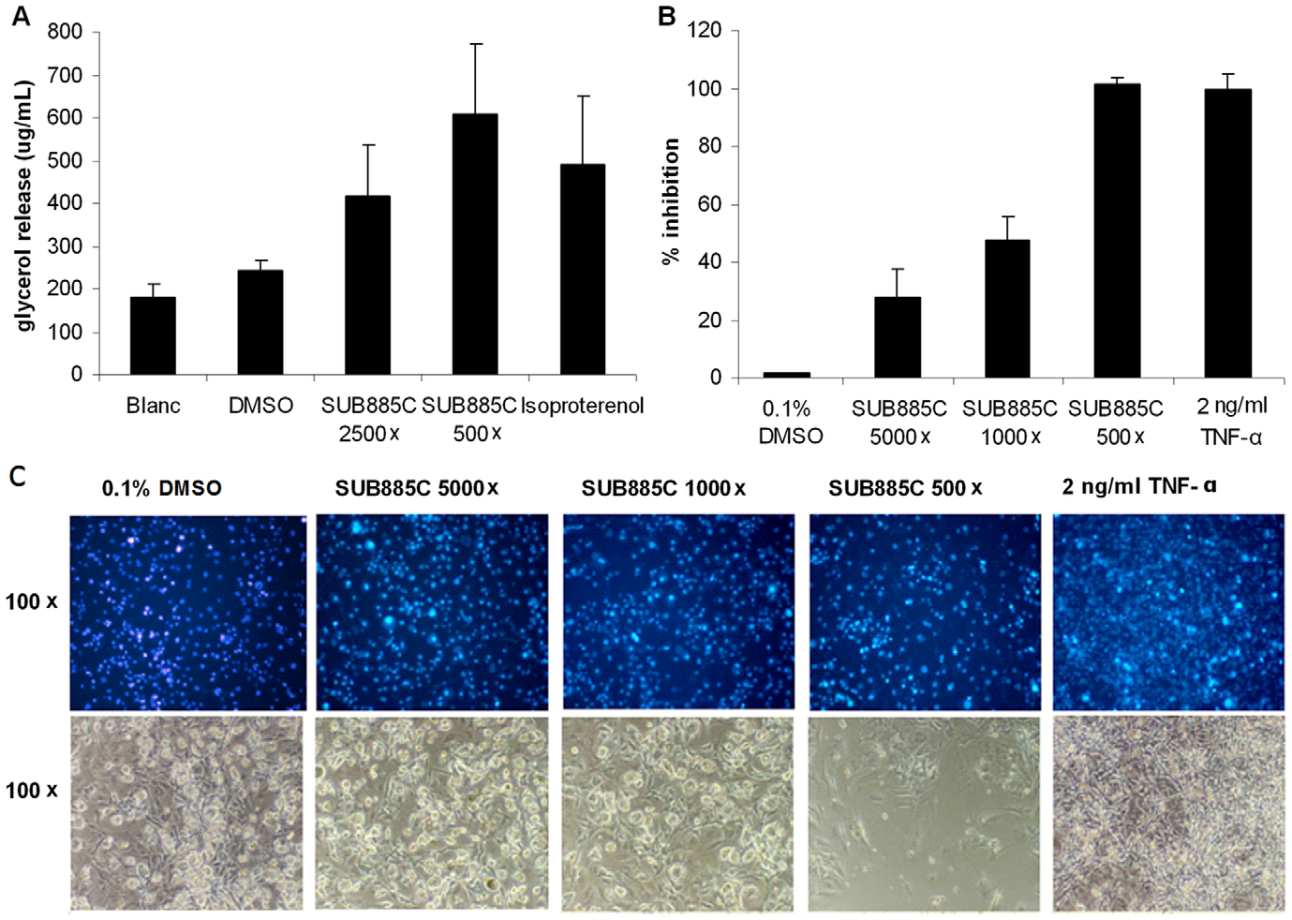
Adipolytic and adipogenic activities of SUB885C in 3T3-L1 adipocytes. (A) Glycerol release as the result of adipolysis in 3T3-L1 adipocytes after incubation with SUB885C (500× and 2500× diluted) and 20 µM isoproterenol dissolved in DMSO with 1% as final concentration. (B) Inhibition of adipose conversion in differentiating 3T3-L1 adipocytes by SUB885C. (C) Morphological analysis of the differentiated 3T3-L1 adipocytes. The cells were treated with 20 µg/ml DAPI (top row), which stained the nuclei of the cells and gave an indication of cell numbers. The phase contrast of the microscope showed the outlines of the cells (bottom row) and gave an overview of cell density and morphology. The magnification is on the left.

To investigate SUB885C's effect on adipolysis, the release of glycerol in the medium was measured after incubation of 3T3-L1 adipocytes with SUB885C. The positive control isoproterenol, a non-selective agonist of the β-adrenergic receptors, is known to increase the hydrolysis of TG [22], [35] and to stimulate adipolysis in 3T3-L1 adipocytes [22] (Figure 4A). When at a 500× dilution, SUB885C extract caused a 22% higher glycerol release as compared to isoproterenol at a concentration of 20 µM. When SUB885C extract was 2500× diluted, glycerol release was 20% lower than 20 µM isoproterenol, yet still higher than DMSO and Blank.

The effect of SUB885C on lipid accumulation in 3T3-L1 adipocytes was investigated during their differentiation process. As positive control the cytokine tumor necrosis factor-α (TNF-α) was used, which interferes with adipocyte differentiation [22]. SUB885C was found to dose-dependently inhibit *in vitro* adipogenesis (Figure 4B). At 5000× dilution there was 30% lipid inhibition in the cells as compared to the control (0.1% DMSO). At 1000× dilution there was 45% lipid inhibition and at 500× dilution there was 100% lipid inhibition in the cells. This was similar for TNF-α at a concentration of 2 ng/ml.

The appearance of 3T3-L1 cells cultured for 9 days in the presence of SUB885C extract was shown in Figure 4C. The number of nuclei did not differ much among the 0.1% DMSO control, 500× and 1000× diluted SUB885C extracts. With 500× dilution (top row) there was a decrease in the amount of nuclei, indicating toxicity induced cell loss, which was in line with the phase contrast picture of microscope with the 500× dilution (bottom row). The other dilutions did not show a clear effect on the cell morphology. TNF-α, as a control, did not show any effect on nuclei numbers and a densely packed cell layer. Inhibition of lipid accumulation in 3T3-L1 cells after exposure to SUB885C extract could be clearly seen at 1000× and 500× dilutions.

## Discussion

Causes and consequences of the complex changes in lipid patterns occurring during development of the metabolic syndrome are still only partly understood. Several interconnected processes are deteriorating which implies that in the end multi-target approaches might be more successful than prevention or intervention strategies based on a limited number of surrogate markers. Results of the present study illustrated that a metabolomics-based analysis of the effects of a multi-component preparation can be used to study potential target processes or novel ingredients. Preparations having their origin in other healthcare systems such as CM have been shown to provide interesting starting points [36]–[42]. In a previous study it was found that SUB885C improved insulin sensitivity compared to control in pre-diabetic ApoE*3Leiden mice fed with a high fat diet for 10 weeks [7]. The ApoE*3Leiden.CETP mouse model used in the present study is a cross-bred of the ApoE*3Leiden mouse with mice expressing human CETP [16]. This further shifts the distribution of Cho from HDL to VLDL/LDL, reduces plasma-mediated scavenger receptor class B type I (SR-BI)-dependent Cho efflux, and produces a strong pro-atherogenic condition [43]. The model has shown its value for the evaluation of interventions that reduce plasma lipids or increase HDL-C [14], [15], [17], [34], [44].

**Figure 5:**
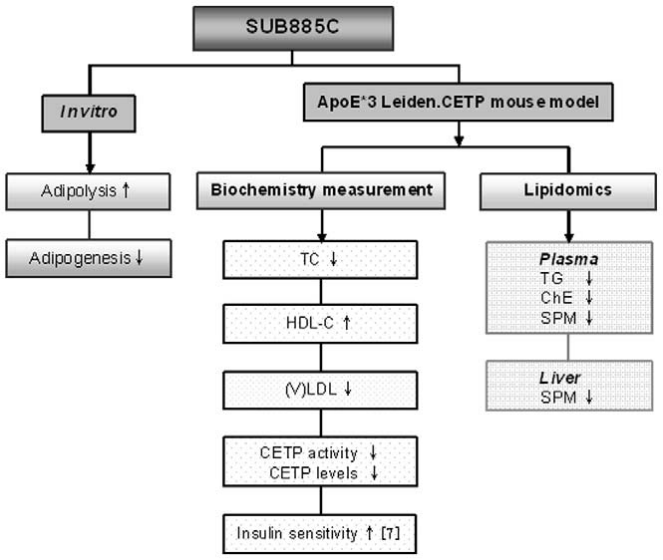
The summary of SUB885C effects. The effects of SUB885C were illustrated both in vitro and in ApoE*3Leiden.CETP mice with biochemical and lipidomics measurements. The improvement for insulin sensitivity was result from Wang et al. [7].

In the present study, SUB885C showed multiple effects to improve metabolic parameters and lipid patterns, which were summarized in figure 5. Specifically, SUB885C treatment induced a increase of plasma HDL-C, which was accompanied with a decrease of (V)LDL levels. Both effects are commonly regarded as anti-atherogenic [14], [17]. Levels and activity of CETP, an important regulator of HDL metabolism, were found to be decreased, which may be related to the reduced levels of VLDL-TG, a substrate for CETP [44]. Taken together, the effects of SUB885C on HDL-C may be due to: 1) a decreased CETP activity and CETP level; 2) a reduced level of (V)LDL, an acceptor for HDL-ChE, which would reduce CETP transfer activity by decreasing the transfer of ChE from HDLs to TG–enriched lipoproteins, and more ChE-enriched HDL particles remain; 3) improved insulin sensitivity. SUB885C significantly reduced plasma TC, which likely reflected the reduction of (V)LDL-C concentrations in the SUB885C treated mice.

During the intervention period total plasma TG levels when measured enzymatically decreased significantly in both the control and the SUB885C groups. At the end of intervention TG level tended to be lower in the SUB885 group compared to that of the control mice, but mainly due to a relatively large within group variance this did not reach significance. With the lipidomics, however, we found a significant reduction of 60% of total plasma TG (*p*<0.01) as compared to that of the controls. This supports our observation that SUB885C treatment induced a TG-lowering effect. SUB885C particularly modulated a wide range of lipids such as TGs, ChEs and SPMs in plasma while in the liver only a few of these lipids were influenced (Table 1). After the correlation analysis of significantly changed lipids both in plasma and liver, only TG-O (50∶0) showed a positive correlation and others not. Instead of a redistribution of lipids between plasma and liver, the effects of SUB885C might relate more to plasma lipoprotein metabolism. Based on the fact that the reduced lipids (i.e. ChE, SPM and TG) in plasma are core lipids of the lipoprotein particle, it seems conceivable that the circulating lipoprotein particles in plasma were reduced by SUB885C due to its influence on lipoprotein regulators such as CETP whose activities are mainly in plasma. This hypothesis is confirmed by the fact that SUB885C (not rimonabant) significantly reduced the level and activity of CETP (Figure 2A) in ApoE*3Leiden.CETP mice.

In Table 2, some of the overall effects of SUB885C were compared with those of the drugs (i.e. rimonabant, atorvastatin, torcetrapib, niacin and tesaglitazer) that were used for modulating plasma lipid profiles obtained in the ApoE*3Leiden.CETP mouse model. Although detailed comparisons cannot be made due to different study designs, effects of SUB885C on these variables appear to be comparable to those of the drugs investigated. Compared with the side-effects induced by these drugs, including psychiatric abnormalities (rimonabant) [45], [46], severe flushing (niacin) [44], headache (atorvastatin), increased risks of cardiovascular morbidity and mortality (torcetrapib) [14], [47], the elevated serum creatinine and associated decreases in glomerular filtration rate (tesaglitazer) [48]; the reported side-effects of the active compounds in SUB885C are scarce. Only herbs containing anthraquinones (i.e. emodin and chrysophanol) were reported to cause diarrhea [12]. Thus, SUB885C might have a higher benefit-risk ratio than other therapeutic interventions.

**Table 2:** Effects on some lipid parameters of SUB885C and existing drugs in the ApoE*3Leiden.CETP female mice model.

	SUB885C	Rimonabant [34]	Atorvastatin [15]	Torcetrapib [14]	Niacin [44]	Tesaglitazer [17]
**Mice age**	6–10 wk	6–10 wk			12 wk	18 wk
**Run-in diet**	4 wk	4 wk	2 wk	4 wk	3 wk	11 wk: 0.3% w/w
(cholesterol)	0.2% w/w	0.2% w/w	0.1% w/w	0.25% w/w	0.1% w/w	4 wk: 0.1%w/w
**Study diet**	same as run-in diet	same as run-in diet	same as run-in diet	same as run-in diet	same as run-in diet	0.1% w/w
(cholesterol)						
**Study period**	4 wk	4 wk	6 wk	14 wk	3 wk	8 wk
**TC**	−49%	−24%	−33%	−20%	−44–68%*	−55%
**TG**	−41% (NS)	−43% (NS)	NS		−57–77%*	−71%
**HDL-C**	+39%	+12% (NS)	+52%	+30%	+77–87%*	+38%
**(V)LDL-C**	−7%	−33%	−88%	−26%	−52–79%*	−80%
**CETP level**	−31%	−4% (NS)	−29% (NS)	+33%	−24–45%*	−42%
**CETP activity**	−74%	−22% (NS)	−36%	−73%	−24–52%*	−56%

Basic diet for ApoE*3Leiden.CETP female mice during run-in and study period: Western type diet with 15% w/w fat plus different content of cholesterol.

NS: no significance.

*Dependent on different doses of Niacin.

In contrast to rimonabant, SUB885C did not reduce body weight or food intake during the 4-week intervention period. Rimonabant is a CB1 inverse agonist, which has been developed and briefly marketed for weight management and improvement of symptoms of the metabolic syndrome. Shortly after its introduction in Europe it was withdrawn because of its central side-effects such as depression and other psychiatric abnormalities [45], [46]. It is now commonly assumed that its action on the central CB1 receptors produces a relatively rapid but transient decrease of appetite [49]. In our study this was observed as a sharp dip in food intake around day 2 (Figure 1). In addition, rimonabant acts on peripheral CB1 receptors leading to a more sustained reduction of body weight and beneficial effects on a number of symptoms of the metabolic syndrome [50]–[52]. Binding studies using a cell membrane preparation expressing the human cannabinoid CB1 receptor showed that a SUB885C total extract was able to displace bound radioactive ligand 3H-CP55940 (data not shown). This could indicate that one or more compounds present in the mixture have affinity for CB1 receptors. However, more data are needed to confirm this. The lack of the effect of SUB885C on body weight and food intake at least suggests that there is no dominating overall effect on the central or peripheral regulation of food-intake or energy regulation.

The *in vitro* results showed that SUB885C is able to stimulate lipolysis and inhibit adipogenesis in 3T3-L1 cells. At this stage it is difficult to speculate on a possible mechanism and its relevance for the overall *in vivo* effects of the preparation since there are several compounds classes that produce such effects, including, but not limited to CB1 blockers [22], [42], [53].

Further studies are needed to reveal the pathways and processes modulated by SUB885C in more detail. Effects of the individual active components in SUB885C have been studied. For example, extracts of Fructus crataegi (Hawthorn berries) are used in CM for treatment of several cardiovascular problems and have been reported to possess anti-inflammatory properties and to modulate mitochondrial functioning [8], [11]. Flos Rosae rugosae (rose flower) extract has been reported to increase the activities of antioxidant enzymes and to reduce lipid peroxidation [10]. Radix et Rhizoma Rhei (rhubarb root) and Radix Glycyrrhizae (licorice root) have been reported to produce inhibition of fatty acid synthase, thus contributing to weight reduction [12]. Radix et Rhizoma Rhei contains emodin which is known as an inhibitor of 11 β-hydroxysteroid dehydrogenase type 1 and has been shown to ameliorate metabolic disorders in diet-induced obese mice [9]. This enzyme is now receiving considerable interest as a pharmacological target in metabolic syndrome [49]. Folium apocyni is obtained from leaves of Apocynum venetum L. (Venetian dogbane) and used in CM to prepare herbal teas against various cardiovascular and other problems.

In conclusion, our study has shown that a CM principle-based multi-components preparation is able to produce anti-atherogenic changes in lipid spectra of the ApoE*3Leiden.CETP mouse model, which are comparable to those obtained with compounds belonging to known drug classes. Our data also illustrate the power of lipidomics in unraveling effects in detail and to help finding new targets or ingredients. These findings can be used to develop new preparations at the nutrition-pharma interface that can be used to prevent metabolic syndrome or ameliorate its first symptoms. Forthcoming studies should include dose-titrations and studies on lipid fluxes in human volunteers.

## Supporting Information

Table S1 **Thirty lipids contributed most to discriminate between control and SUB885C treated mice in plasma and liver.**
(DOC)

Table S2 **Lipid molecular species are significantly influenced in plasma upon SUB885C treatment as compared to non-treated controls.**
(DOC)

Table S3 **Lipid molecular species are significantly influenced in liver tissue upon SUB885C treatment as compared to non-treated controls.**
(DOC)
