# Supplementary material for: Lipidomics Reveals Multiple Pathway Effects of a Multi-Components Preparation on Lipid Biochemistry in ApoE*3Leiden.CETP Mice
Source: PLoS One. 2012 Jan 23;7(1):e30332. doi: 10.1371/journal.pone.0030332 (PMC3264613; doi:10.1371/journal.pone.0030332)
Supplement: Table S1 — Thirty lipids contributed most to discriminate between control and SUB885C treated mice in plasma and liver. (DOC) [file pone.0030332.s001.doc]

**Table S1. Thirty lipids contributed most to discriminate between control and SUB885C treated mice in plasma and liver**

| **Ranking ^*^** | **Plasma lipids** | **Liver lipids** |
| --- | --- | --- |
| 1 | ChE (18:1) | PC (34:3) |
| 2 | ChE (18:1)-Dimeric | LPC (16:0) |
| 3 | ChE (18:2) | SPM (20:0) |
| 4 | PC (38:6) | TG (52:6) |
| 5 | ChE (18:3)-Dimeric | TG (52:3) |
| 6 | ChE (18:3) | SPM (15:0) |
| 7 | PC (36:6) | PC (40:5) |
| 8 | PC (38:4) | PC (36:5) |
| 9 | PC (40:5) | TG (52:2) |
| 10 | PE-O (38:5) | TG (50:2) |
| 11 | PC-O (42:6) | ChE (22:6) |
| 12 | SPM (24:2) | TG (50:1) |
| 13 | ChE (18:2)-Dimeric | PE (38:6) |
| 14 | PE (38:2) | SPM (22:1) |
| 15 | PE (38:4) | PC (40:8) |
| 16 | SPM (14:0) | TG (50:3) |
| 17 | SPM (22:1) | PC (36:1) |
| 18 | SPM (18:1) | TG (50:0) |
| 19 | SPM (18:0) | PC (38:6) |
| 20 | SPM (24:1) | LPE (20:4) |
| 21 | PC (40:4) | PC (38:3) |
| 22 | SPM (15:0) | PC-O (38:4) |
| 23 | SPM (22:0) | TG (52:1) |
| 24 | PC (40:7) | PC (40:7) |
| 25 | PE (36:3) | PE (34:1) |
| 26 | SPM (23:0) | PC-O (34:1) |
| 27 | TG (54:5) | TG (52:5) |
| 28 | TG (46:1) | SPM (22:0) |
| 29 | TG (50:3) | PE (38:3) |
| 30 | SPM (20:0) | PC-O (36:4) |

**^*^** The ranking is calculated based on the coefficients of PLS-DA regression vector.

Eight gray highlighted lipids are similar lipids present in both plasma and liver samples for the discrimination between control and treated mice.
