# Supplementary material for: Lipidomics Reveals Multiple Pathway Effects of a Multi-Components Preparation on Lipid Biochemistry in ApoE*3Leiden.CETP Mice
Source: PLoS One. 2012 Jan 23;7(1):e30332. doi: 10.1371/journal.pone.0030332 (PMC3264613; doi:10.1371/journal.pone.0030332)
Supplement: Table S2 — Lipid molecular species are significantly influenced in plasma upon SUB885C treatment as compared to non-treated controls. (DOC) [file pone.0030332.s002.doc]

**Table S2. Lipid molecular species are significantly influenced in plasma upon SUB885C treatment** as compared to non-treated controls

|  |  |  | **SUB885C** |  |  |
| --- | --- | --- | --- | --- | --- |
| **Lipid species** | **control** | **SUB885C** | **vs. control** | ***p* value** | **Up (**↑**) or** |
|  | **(mean ± SD)** | **(mean ± SD)** | **change (%)** |  | **down (**↓**)** |
| LPC (14:0) | 0.0008 ± 0.0001 | 0.0007 ± 0.0001 | 15 | **<0.05** | ↓ |
| LPC (18:2) | 0.14 ± 0.01 | 0.12 ± 0.01 | 16 | **<0.01** | ↓ |
| LPC-O (18:1) | 0.0033 ± 0.0008 | 0.0024 ± 0.0003 | 28 | <0.05 | ↓ |
| PC (32:0) | 0.0034 ± 0.0004 | 0.0029 ± 0.0003 | 16 | **<0.01** | ↓ |
| PC (36:5) | 0.0006 ± 0.0001 | 0.0007 ± 0.0001 | 34 | <0.05 | ↓ |
| PC (38:2) | 0.51 ± 0.06 | 0.36 ± 0.04 | 29 | **<0.001** | ↓ |
| PC (38:4) | 0.020 ± 0.003 | 0.029 ± 0.007 | 43 | **<0.05** | ↑ |
| PC (38:6) | 0.010 ± 0.002 | 0.014 ± 0.003 | 31 | <0.05 | ↑ |
| PC (40:7) | 0.0025 ± 0.0003 | 0.0030± 0.0004 | 22 | **<0.05** | ↑ |
| PC-O (34:3) | 0.00006 ± 0.00001 | 0.00005 ± 0.00001 | 17 | <0.05 | ↓ |
| PC-O (38:7) | 0.00008 ± 0.00003 | 0.00004 ± 0.00002 | 44 | **<0.01** | ↓ |
| PE (34:2) | 0.014 ± 0.003 | 0.008 ± 0.003 | 42 | **<0.01** | ↓ |
| SPM (14:0) | 0.00037 ± 0.00004 | 0.00026 ± 0.00002 | 31 | **<0.001** | ↓ |
| SPM (16:0) | 0.0046 ± 0.0003 | 0.0042 ± 0.0004 | 11 | <0.05 | ↓ |
| SPM (16:1) | 0.15 ± 0.02 | 0.10 ± 0.02 | 31 | **<0.001** | ↓ |
| SPM (22:0) | 0.27 ± 0.03 | 0.19 ± 0.05 | 31 | **<0.001** | ↓ |
| SPM (22:1) | 0.0009 ± 0.0001 | 0.0010 ± 0.0001 | 17 | <0.05 | ↑ |
| SPM (23:0) | 0.13 ± 0.01 | 0.09 ± 0.01 | 28 | **<0.001** | ↓ |
| SPM (23:1) | 0.00031 ± 0.00004 | 0.00027 ± 0.00002 | 13 | <0.05 | ↓ |
| SPM (24:0) | 0.0095 ± 0.0008 | 0.0081 ± 0.0007 | 15 | **<0.001** | ↓ |
| SPM (24:1) | 0.0044 ± 0.0004 | 0.0034 ± 0.0003 | 22 | **<0.001** | ↓ |
| SPM (24:2) | 0.0018 ± 0.0001 | 0.0015 ± 0.0002 | 17 | **<0.01** | ↓ |
| ChE (18:1) | 0.30 ± 0.04 | 0.18 ± 0.02 | 40 | **<0.001** | ↓ |
| ChE (18:2) | 2.9 ± 0.6 | 1.2 ± 0.3 | 57 | **<0.001** | ↓ |
| ChE (18:3) | 0.0011 ± 0.0003 | 0.0004 ± 0.0001 | 65 | **<0.001** | ↓ |
| ChE (20:4) | 0.14 ± 0.03 | 0.10 ± 0.01 | 29 | <0.05 | ↓ |
| ChE (22:6) | 0.015 ± 0.004 | 0.009 ± 0.002 | 38 | **<0.001** | ↓ |
| ChE (18:1)-Dimeric | 12 ± 2 | 7± 1 | 43 | **<0.01** | ↓ |
| ChE (18:2)-Dimeric^*^ | 1.6 ± 0.5 | 0.7 ± 0.2 | 55 | **<0.001** | ↓ |
| ChE (18:3)-Dimeric | 0.0045 ± 0.0015 | 0.0011 ± 0.0005 | 75 | **<0.001** | ↓ |
| ChE (22:6)-Dimeric | 0.02 ± 0.01 | 0.007 ± 0.003 | 65 | **<0.01** | ↓ |
| TG (46:0) | 0.033 ± 0.004 | 0.027 ± 0.004 | 19 | **<0.05** | ↓ |
| TG (46:1) | 0.013 ± 0.004 | 0.007 ± 0.002 | 47 | **<0.01** | ↓ |
| TG (48:0) | 0.17 ± 0.06 | 0.06 ± 0.03 | 63 | **<0.01** | ↓ |
| TG (48:1) | 0.008 ± 0.003 | 0.003 ± 0.002 | 61 | **<0.01** | ↓ |
| TG (48:2) | 0.10 ± 0.03 | 0.04 ± 0.02 | 62 | **<0.01** | ↓ |
| TG (50:0) | 0.05 ± 0.02 | 0.015 ± 0.010 | 70 | **<0.01** | ↓ |
| TG (50:1) | 0.16 ± 0.07 | 0.06 ± 0.03 | 65 | **<0.01** | ↓ |
| TG (50:2) | 0.11 ± 0.04 | 0.05 ± 0.02 | 59 | **<0.01** | ↓ |
| TG (50:3) | 0.020 ± 0.006 | 0.009 ± 0.004 | 54 | **<0.01** | ↓ |
| TG (50:4) | 0.017 ± 0.004 | 0.011 ± 0.003 | 38 | <0.05 | ↓ |
| TG (52:0) | 0.06 ± 0.03 | 0.02 ± 0.01 | 68 | **<0.01** | ↓ |
| TG (52:1) | 0.22 ± 0.11 | 0.07 ± 0.04 | 70 | **<0.01** | ↓ |
| TG (52:2) | 1.0 ± 0.4 | 0.4 ± 0.2 | 59 | **<0.01** | ↓ |
| TG (52:3) | 0.31 ± 0.11 | 0.14 ± 0.07 | 54 | **<0.05** | ↓ |
| TG (52:4) | 0.7 ± 0.3 | 0.3 ± 0.1 | 64 | **<0.01** | ↓ |
| TG (52:5) | 0.004 ± 0.001 | 0.0021 ± 0.0006 | 51 | **<0.01** | ↓ |
| TG (52:6) | 0.0025 ± 0.0008 | 0.0011 ± 0.0006 | 57 | **<0.01** | ↓ |
| TG (54:0) | 0.025 ± 0.012 | 0.008 ± 0.006 | 67 | **<0.05** | ↓ |
| TG (54:1) | 0.12 ± 0.07 | 0.04 ± 0.03 | 68 | **<0.05** | ↓ |
| TG (54:2) | 0.32 ± 0.17 | 0.11 ± 0.06 | 66 | **<0.01** | ↓ |
| TG (54:3) | 0.9 ± 0.4 | 0.4 ± 0.2 | 58 | **<0.01** | ↓ |
| TG (54:4) | 0.18 ± 0.08 | 0.08 ± 0.04 | 57 | **<0.01** | ↓ |
| TG (54:5) | 0.05 ± 0.02 | 0.021 ± 0.009 | 55 | **<0.05** | ↓ |
| TG (54:6) | 0.012 ± 0.004 | 0.007 ± 0.002 | 47 | **<0.05** | ↓ |
| TG (54:7) | 0.0012 ± 0.0004 | 0.0007 ± 0.0002 | 40 | <0.05 | ↓ |
| TG (56:0) | 0.0028 ± 0.0013 | 0.0011 ± 0.0007 | 61 | **<0.05** | ↓ |
| TG (56:1) | 0.012 ± 0.006 | 0.004 ± 0.003 | 64 | **<0.05** | ↓ |
| TG (56:2) | 0.021 ± 0.010 | 0.008 ± 0.004 | 61 | **<0.01** | ↓ |
| TG (56:3) | 0.10 ± 0.05 | 0.04 ± 0.02 | 55 | **<0.05** | ↓ |
| TG (56:4) | 0.08 ± 0.03 | 0.03 ± 0.02 | 55 | **<0.01** | ↓ |
| TG (56:5) | 0.12 ± 0.04 | 0.06 ± 0.03 | 48 | <0.05 | ↓ |
| TG (56:6) | 0.05 ± 0.02 | 0.03 ± 0.01 | 49 | **<0.05** | ↓ |
| TG (56:7) | 0.37 ± 0.16 | 0.15 ± 0.06 | 60 | **<0.01** | ↓ |
| TG (56:8) | 0.0043 ± 0.0017 | 0.0022 ± 0.0006 | 48 | **<0.05** | ↓ |
| TG (58:1) | 0.004 ± 0.002 | 0.002 ± 0.001 | 64 | **<0.05** | ↓ |
| TG (58:2) | 0.004 ± 0.002 | 0.0013 ± 0.0009 | 65 | **<0.05** | ↓ |
| TG (58:3) | 0.006 ± 0.003 | 0.002 ± 0.001 | 59 | **<0.01** | ↓ |
| TG (58:4) | 0.005± 0.002 | 0.003 ± 0.001 | 51 | **<0.01** | ↓ |
| TG (58:5) | 0.016 ± 0.007 | 0.008± 0.005 | 49 | <0.05 | ↓ |
| TG (58:6) | 0.016 ± 0.006 | 0.008 ± 0.004 | 51 | **<0.01** | ↓ |
| TG (58:7) | 0.026 ± 0.009 | 0.013 ± 0.007 | 50 | **<0.01** | ↓ |
| TG (58:8) | 0.015 ± 0.005 | 0.007 ± 0.002 | 54 | **<0.01** | ↓ |
| TG (58:9) | 0.09 ± 0.04 | 0.04 ± 0.01 | 55 | **<0.05** | ↓ |
| TG (60:1) | 0.002 ± 0.001 | 0.0007 ± 0.0005 | 63 | <0.05 | ↓ |
| TG (60:2) | 0.002 ± 0.001 | 0.0007 ± 0.0005 | 67 | **<0.05** | ↓ |
| TG (60:3) | 0.0011 ± 0.0006 | 0.0004 ± 0.0002 | 67 | **<0.01** | ↓ |
| TG-O (50:0) | 0.011 ± 0.006 | 0.003 ± 0.002 | 70 | **<0.05** | ↓ |
| TG-O (50:1) | 0.0035 ± 0.0016 | 0.0011 ± 0.0007 | 70 | **<0.05** | ↓ |
| TG-O (50:2) | 0.0008 ± 0.0003 | 0.0003 ± 0.0002 | 67 | **<0.05** | ↓ |
| TG-O (52:0) | 0.016 ± 0.008 | 0.005 ± 0.004 | 71 | **<0.05** | ↓ |
| TG-O (52:1) | 0.0037 ± 0.0017 | 0.0011 ± 0.0008 | 70 | **<0.05** | ↓ |
| TG-O (56:2) | 0.008 ± 0.004 | 0.003 ± 0.002 | 70 | <0.05 | ↓ |
| TG-O (56:3) | 0.002 ± 0.001 | 0.0007± 0.0005 | 69 | **<0.05** | ↓ |
| TG-O (58:2) | 0.006 ± 0.004 | 0.002 ± 0.001 | 69 | <0.05 | ↓ |
| TG-O (58:3) | 0.0023 ± 0.0012 | 0.0008 ± 0.0006 | 67 | <0.05 | ↓ |

*p* values correspond to the mean difference between the SUB885C group and the control group.

*Note*: lipids with *p* values marked in bold mean those remain significant after MTC.

LPC-O: ether LPC; PC-O: ether PC; TG-O: ether TG

* Homogeneity of variance assumption is deviated of this lipid. Data was log transformed and the mean ± SD was shown as the normal data value for a reference.
