# Supplementary material for: Lipidomics Reveals Multiple Pathway Effects of a Multi-Components Preparation on Lipid Biochemistry in ApoE*3Leiden.CETP Mice
Source: PLoS One. 2012 Jan 23;7(1):e30332. doi: 10.1371/journal.pone.0030332 (PMC3264613; doi:10.1371/journal.pone.0030332)
Supplement: Table S3 — Lipid molecular species are significantly influenced in liver tissue upon SUB885C treatment as compared to non-treated controls. (DOC) [file pone.0030332.s003.doc]

**Table S3. Lipid molecular species are significantly influenced in liver tissue upon SUB885C treatment as compared to non-treated controls**

|  |  |  | **SUB885C** |  |  |
| --- | --- | --- | --- | --- | --- |
| **Lipid species** | **control** | **SUB885C** | **vs. control** | ***p* value** | **Up (**↑**) or** |
|  | **(mean ± SD)** | **(mean ± SD)** | **change (%)** |  | **down (**↓**)** |
| LPC (18:1) | 0.34 ± 0.04 | 0.29± 0.05 | 12 | <0.05 | ↓ |
| LPC (18:2) | 0.11 ± 0.02 | 0.08 ± 0.02 | 28 | <0.01 | ↓ |
| PC (32:0) | 0.29 ± 0.02 | 0.26 ± 0.02 | 10 | <0.05 | ↓ |
| PC (36:5) | 0.55 ± 0.06 | 0.64 ± 0.08 | 16 | <0.05 | ↑ |
| PC-O (38:5)^*^ | 0.33 ± 0.04 | 0.25 ± 0.02 | 21 | <0.05 | ↓ |
| PC-O (40:6) | 0.025 ± 0.002 | 0.020 ± 0.004 | 33 | <0.05 | ↓ |
| PE (34:2) | 0.25 ± 0.05 | 0.20 ± 0.03 | 20 | <0.05 | ↓ |
| PE (36:2) | 0.76 ± 0.11 | 0.63 ± 0.06 | 17 | <0.05 | ↓ |
| PE (36:3) | 0.53 ± 0.13 | 0.39 ± 0.06 | 26 | <0.05 | ↓ |
| SPM (22:1) | 0.63 ± 0.05 | 0.51 ± 0.05 | 19 | <0.01 | ↓ |
| SPM (24:1)^*^ | 0.86 ± 0.11 | 0.68 ± 0.07 | 21 | <0.05 | ↓ |
| ChE (18:2) | 0.29 ± 0.06 | 0.16 ± 0.05 | 44 | **<0.01** | ↓ |
| ChE (20:4) | 0.14 ± 0.06 | 0.07 ± 0.01 | 50 | <0.01 | ↓ |
| TG (50:1) | 1.4 ± 0.4 | 1.8 ± 0.3 | 30 | <0.05 | ↑ |
| TG (54:0) | 0.04 ± 0.01 | 0.024 ± 0.004 | 50 | <0.01 | ↓ |
| TG-O (50:0) | 0.038 ± 0.007 | 0.026 ± 0.005 | 30 | **<0.01** | ↓ |
| TG-O (50:1) | 0.065 ± 0.008 | 0.053 ± 0.004 | 29 | <0.01 | ↓ |
| TG-O (50:2) | 0.016 ± 0.002 | 0.013 ± 0.002 | 50 | <0.05 | ↓ |
| TG-O (52:1) | 0.11 ± 0.02 | 0.077 ± 0.008 | 28 | **<0.001** | ↓ |
| TG-O (52:2) | 0.12 ± 0.02 | 0.10 ± 0.01 | 17 | <0.01 | ↓ |
| TG-O (58:1) | 0.015 ± 0.003 | 0.009 ± 0.002 | 40 | <0.01 | ↓ |
| TG-O (58:2)^*^ | 0.034 ± 0.006 | 0.024 ± 0.005 | 33 | <0.05 | ↓ |

*p* values correspond to the mean difference between the SUB885C group and the control group.

*Note*: lipids with *p* values marked in bold mean those remain significant after MTC.

PC-O: ether PC; TG-O: ether TG

* Homogeneity of variance assumption is deviated of this lipid. Data was log transformed and the mean ± SD was shown as the normal data value for a reference.
